# *Plasmodium falciparum* infected erythrocytes can bind to host receptors integrins αVβ3 and αVβ6 through DBLδ1_D4 domain of PFL2665c PfEMP1 protein

**DOI:** 10.1038/s41598-018-36071-2

**Published:** 2018-12-14

**Authors:** Olga Chesnokov, Jordan Merritt, Sergey O. Tcherniuk, Neta Milman, Andrew V. Oleinikov

**Affiliations:** 1 Charles E. Schmidt College of Medicine, Department of Biomedical Science, Florida Atlantic University, Boca Raton, FL USA; 2 Seattle Biomedical Research Institute, Seattle, WA USA

## Abstract

Major complications and mortality from *Plasmodium falciparum* malaria are associated with cytoadhesion of parasite-infected erythrocytes (IE). The main parasite ligands for cytoadhesion are members of the *P*. *falciparum* erythrocyte membrane protein 1 (PfEMP1) family. Interactions of different host receptor-ligand pairs may lead to various pathological outcomes, like placental or cerebral malaria. It has been shown previously that IE can bind integrin αVβ3. Using bead-immobilized PfEMP1 constructs, we have identified that the PFL2665c DBLδ1_D4 domain binds to αVβ3 and αVβ6. A parasite line expressing PFL2665c binds to surface-immobilized αVβ3 and αVβ6; both are RGD motif-binding integrins. Interactions can be inhibited by cyloRGDFV peptide, an antagonist of RGD-binding integrins. This is a first, to the best of our knowledge, implication of a specific PfEMP1 domain for binding to integrins. These host receptors have important physiological functions in endothelial and immune cells; therefore, these results will contribute to future studies and a better understanding, at the molecular level, of the physiological outcome of interactions between IE and integrin receptors on the surface of host cells.

## Introduction

Cytoadhesion of parasite-infected erythrocytes (IE) to endothelium and to immune cells plays a major role in complications and mortality from *Plasmodium falciparum* malaria^1^. At present, more than a dozen human receptors have been identified that bind to IE. Most of them are expressed on endothelia and include CD36 (cluster of differentiation 36); ICAM-1 (intercellular adhesion molecule-1); EPCR (endothelial protein C receptor); CSA (chondroitin sulfate A, in placenta); PECAM-1 (platelet endothelial cell adhesion molecule-1, CD31); TSP-1 (thrombospondin-1); non-immune IgM (in plasma); VCAM-1 (vascular cell adhesion molecule-1); integrin αVβ3; CR1 (complement receptor 1); heparan sulfate; E-selectin; and P-selectin (reviewed in^2–4^). The main parasite ligands for cytoadhesion are members of the *P*. *falciparum* erythrocyte membrane protein 1 (PfEMP1) family. However, detailed characterization of PfEMP1 domain-receptor pairs including the association of adhesion with specific domain sub-classes has been performed for only CD36^5,6^, ICAM-1^7,8^, CSA^9,10^, IgM^11,12^, and EPCR^13,14^ (reviewed in^4,15^). This gap in knowledge significantly restricts our understanding of malaria pathophysiology and the development of treatments against severe malaria. The recent discovery of EPCR as a novel malaria receptor as well as the identification and characterization of EPCR-binding PfEMP1 interacting domains^13^ and their involvement in severe malaria^13,16^ clearly demonstrate how identification of novel host receptors may influence studies on malaria pathology. Characterization of other IE-host receptor interactions at the molecular level will create a more complete picture of malaria parasite-host interactions and may have an impact on understanding malaria pathophysiology.

It has been shown previously that IE can bind integrin αVβ3^17^. In line with this finding, analysis of seven *P*. *falciparum* genomes revealed potential binding sites for Arg-Glu-Asp (RGD)-binding integrins in PfEMP1 proteins, pointing to the DBLα0 class of domains as the most likely targets^18^. Twenty-three RGD motifs in the NF54 genome are present in 21 DBL domain and 1 interdomain region; only one domain, PFL2665c DBLδ1_D4, contains two RGD sequences. We decided to test this delta sub-class domain^18^, along with ten domains not containing a RGD sequence, and four DBL domains containing a single RGD sequence (3 of which were of the α0 class) for αVβ3 integrin binding. Since all domains with RGD sequences were DBL domains followed by CIDR domains, which may organize a structural-functional unit, we initially tested these domains as DBL-CIDR tandem constructs. Only PFL2665c DBLδ1_D4-CIDRβ1_D5 domain tandem demonstrated integrin binding and was further studied using various *in vitro* approaches including live IE expressing PFL2665c protein. As a result, this work identified several individual DBL domains as integrin αVβ3- and αVβ6-binding functional units with the results confirmed with live IE expressing PFL2665c protein.

## Results and Discussion

Identification of novel host receptors that serve as ligands for IE is an important area of malaria studies. Recent examples include: a) identification of a novel interaction between EPCR and specific PfEMP1 domains and implication of this interaction in severe malaria^13^, including potential importance of EPCR- and ICAM1-binding PfEMP1 domain tandem presence within the same protein for cerebral malaria^14^; b) discovery that another variable surface protein of *P*. *falciparum*, a member of the rifin family, interacts with leucocyte immunoglobulin-like receptor B1 and downregulates activation of immune cells^19^. These examples clearly demonstrate that our knowledge about host-parasite interactions is still limited and identification of novel receptor-domain pairs is essential for studies of malaria pathophysiology.

Integrins are an important class of cell surface receptors involved in a wealth of physiological functions in endothelial and immune cells^20^. Previously, it was shown that IE may interact with αVβ3 integrin^17^, but no IE ligand or details of this process have been described. The Arg-Glu-Asp (RGD) motif, found in many proteins, is the integrin-binding sequence for a third of integrins including αVβ3. A computational search in seven genomes for the RGD sequence in PfEMP1 proteins, including the NF54 line, found that only DBL, and not CIDR, domains contain this motif^18^. We have found that in NF54 line only one DBL domain, DBLδ1_D4 from PFL2665c, contains two of RGD motifs. The two RGD sequences are located in the loops of the predicted domain secondary structure (Supplementary Fig. 1). The location and surrounding sequence might be important for efficient integrin binding^20^. Therefore, we first tested this domain (in tandem with CIDRβ1_D5), other tandem domains containing only one RGD sequence, and a variety of domains from different domain classes without an RGD sequence for αVβ3 integrin binding. Constructs were selected based on their robust expression in our mammalian expression system^21^ and on CD36 binding by those tandems that contain CD36-binding domains, which indicates their correct folding (Supplementary Fig. 2).

**Figure 1:**
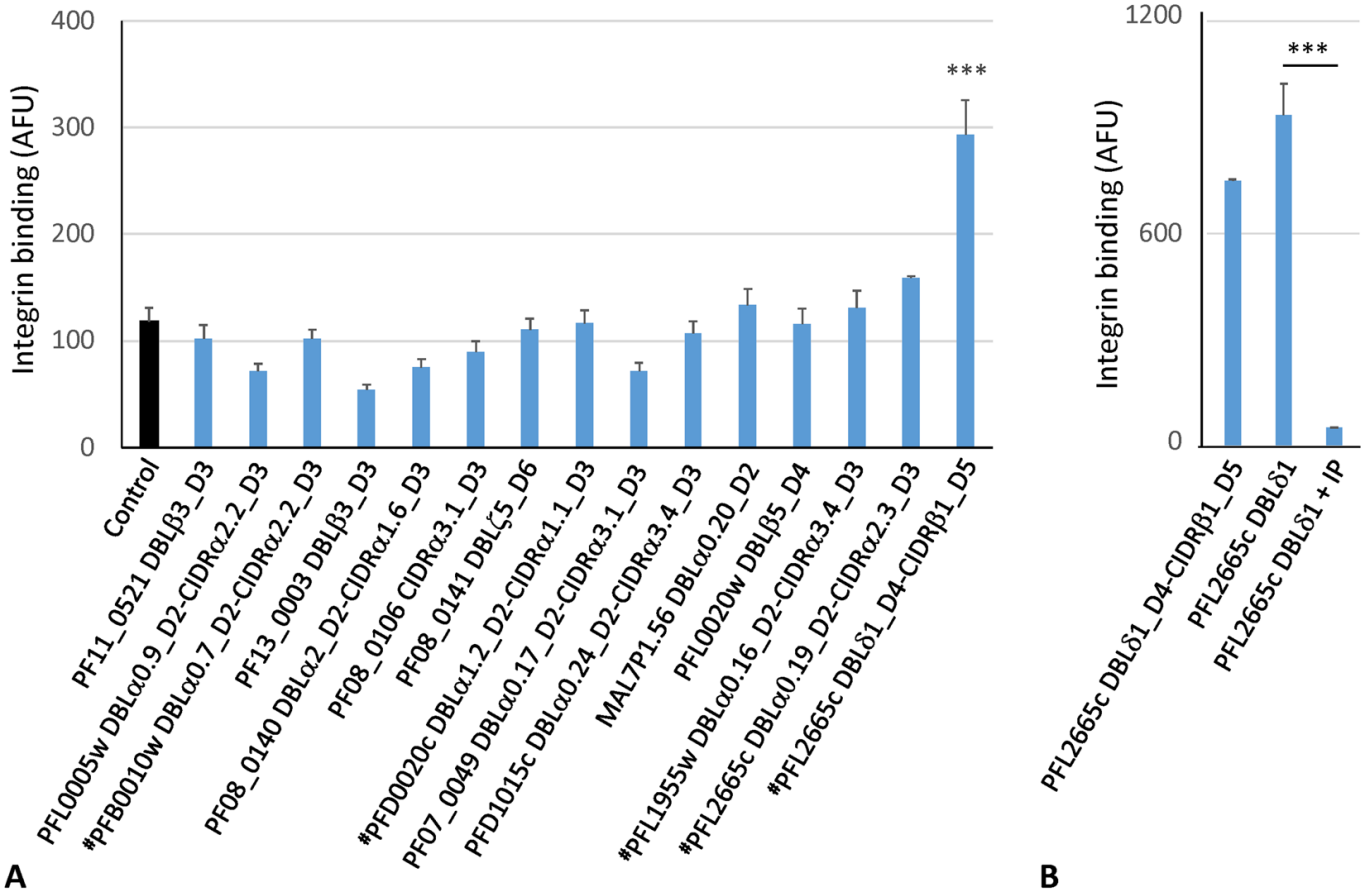
DBLδ1_D4 domain of PFL2665 binds specifically to integrin αVβ3. (**A**) Testing various PfEMP1 constructs immobilized on BioPlex beads for binding to soluble biotinylated integrin at 5 µg/ml. Control is binding to control construct HisAdEx^8,21^. Cut-off is two SD of control. *** indicate p-value < 0.001 for comparison with Control (t-test). (**B**) Binding of integrin to tandem (DBLδ1_D4-CIDRβ1_D5) and single (DBLδ1_D4) PFL2665 domains and inhibition of binding by RGD inhibitory peptide (IP) at 1 μg/ml. AFU, Arbitrary fluorescence units. Binding to control construct HisAdEx was subtracted from binding to domain constructs. Bars represent means of duplicate measurements. Error bars indicate Standard Deviations (SD). P value obtained by Holm-Sidak’s multiple comparisons test (ANOVA). ***p < 0.001. These experiments were repeated at least three times with similar qualitative results.

Our results (Fig. 1a) demonstrated that only DBLδ1_D4-CIDRβ1_D5 tandem from PFL2665c binds αVβ3 integrin. Further, we tested if a single PFL2665c DBLδ1_D4 domain, containing two RGD sequences is able to bind αVβ3 integrin. The results shown in Fig. 1b confirm that the single DBLδ1_D4 domain is as efficient in binding αVβ3 integrin as the tandem DBLδ1_D4-CIDRβ1_D5. We also demonstrated that a known specific inhibitor of αVβ3, the cyclic peptide antagonist cyloRGDFV, could block this interaction. We measured avidity of interaction between αVβ3 integrin and the single DBLδ1_D4 domain using an approach we developed previously for measuring the avidity of interactions between ICAM1 and ICAM1-binding DBLβ3_D4_PF11_0521_ domain using BioPlex beads^22^. The accuracy of our bead method was recently proven by measurement of the almost identical K_D_ value for interaction between ICAM1 and the same DBLβ3_D4_PF11_0521_ domain expressed in similar boundaries using surface plasmon resonance^14^. Using our bead method, the K_D_ of integrin::DBLδ1_D4_PFL2665c_ interactions was found to be 62 ± 4.9 nM (mean ± SEM; Supplementary Fig. 3a). This indicates strong binding, within the range found for binding of CIDR domains to CD36 receptor^6^, which may produce stable association between receptor and PfEMP1 domains that, in turn, may affect physiology of the cells interacting with IE.

**Figure 2:**
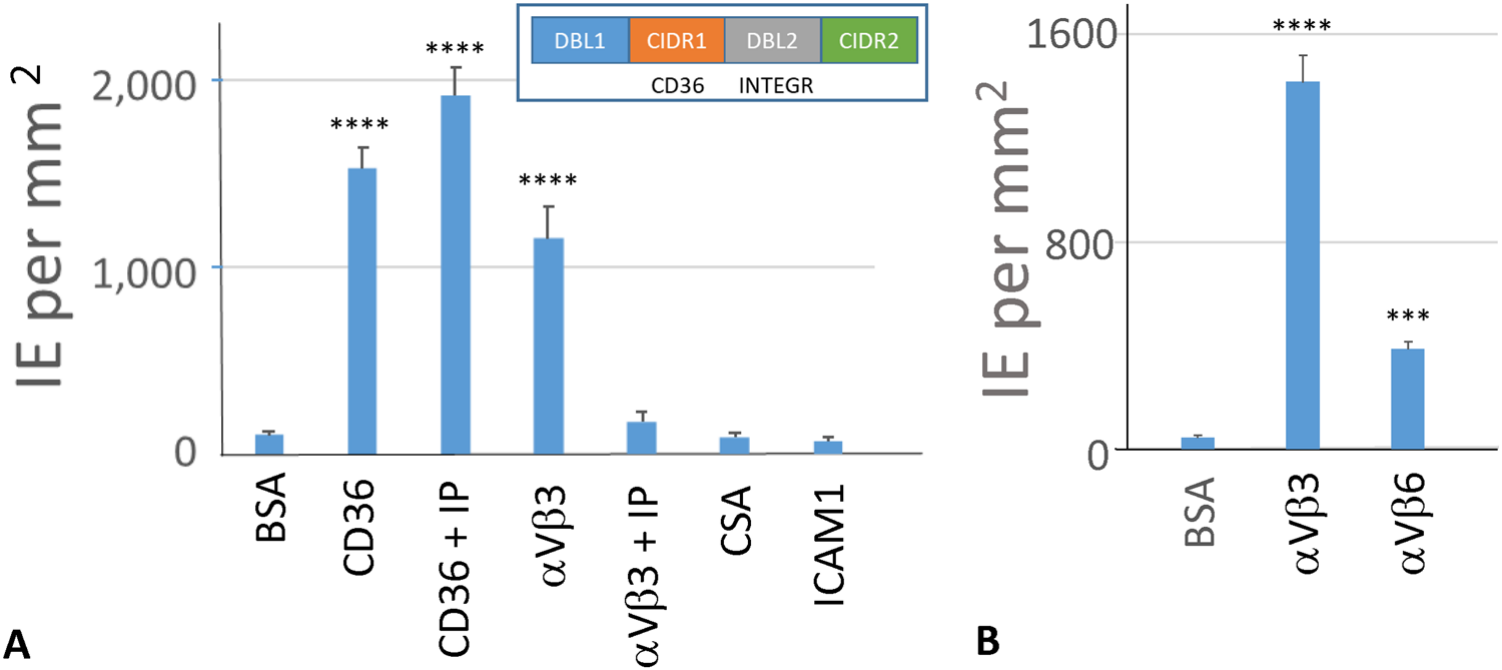
Specific binding of E9 line to integrins and other receptors. (**A**) Binding of E9 line (NF54 genetic background) to surface-immobilized receptors in absence and presence (10 μg/ml) of RGD inhibitory peptide (IP). Receptors CD36, αVβ3, CSA immobilized at 10 μg/ml and ICAM1 at 100 μg/ml concentrations. Culture is at 22% parasitemia, 0.5% hematocrit. Inset – domain structure and binding specificities of the PFL2665c expressed by E9 line. (**B**) Binding of E9 to two surface-immobilized (at 10 μg/ml) RGD-binding integrins. Culture is at 17% parasitemia, 0.5% hematocrit. Binding was measured by counting attached infected erythrocytes in 20–30 microscope fields in two spots of each receptor immobilized on the surface of Petri dish. Bars indicate Means and Error bars indicate Standard Error of Means (SEM). Differences in binding of E9 to each receptor and BSA control were calculated by one-way ANOVA using Holm-Sidak’s multiple comparison tests. ***p < 0.001, ****p < 0.0001. Results of typical experiment are presented. Experiment was repeated at least 3 times with similar qualitative results.

Further, to confirm that IE expressing PFL2665c protein can bind αVβ3 integrin, we used the PFL2665c-expressing E9 parasite line, previously isolated from parent NF54 by limiting dilutions^23^. As PFL2665c also contains CD36-binding CIDRα2.3_D3 domain^24^ (Fig. 2A inset), this line binds to CD36 and αVβ3 integrin but not to CSA, ICAM1, or control protein (BSA) (Fig. 2A). Binding of E9 to αVβ3 is inhibited by soluble αVβ3 integrin (Supplementary Fig. 4), and binding to CD36 is inhibited by soluble CD36 and by binding-inhibitory anti-CD36 mAb FA6-152^25,26^ (Supplementary Fig. 5), confirming specificity of receptor binding. In addition, cyloRGDFV inhibitory peptide (IP) completely inhibits binding of E9 line to αVβ3 but not to CD36 (Fig. 2A), confirming the specificity of the interaction with the integrin receptor through RGD motif. There are several RGD-binding integrins^20^. We have tested and demonstrated binding of the E9 line to recombinant αVβ6 (Fig. 2B), another RGD-binding integrin, as well as binding of bead-immobilized DBLδ1_D4 to αVβ6 with the K_D_ of 56.4 ± 9.8 nM (mean ± SEM; Supplementary Fig. 3b), which is similar to the K_D_ of αVβ3. Both RGD motifs in DBLδ1_D4_PFL2665c_ map to the structural loops^27,28^ using secondary structure prediction^29^ (Supplementary Fig. 1). Moreover, the second RGD is located between two Cys residues, both involved in two disulfide bonds^28^ (not to each other), making a perfect loop with RGD sequence, Cys-RGDKV-Cys. Whether presence of two RGD sequences in one domain or just one RGD in the right context provide measured high avidity to αVβ3 and αVβ6 is not clear at this time.

Two other lines, selected for binding to CSA (CS2 line expressing VAR2CSA PfEMP1^30^) and ICAM-1 (3G8 line expressing VAR1 PfEMP1^31^), do not bind to surface-immobilized αVβ3 (Supplementary Fig. 6). It is interesting that VAR1 protein (GenBank: AAO67411.1) is a rare case when an RGD motif is located in the CIDRγ6 class domain^18^ (amino acid residues 2183–2185) and not in the DBL class domain, a common domain class for RGD motif presence in PfEMP1 proteins. This might be the reason why IE expressing this protein does not bind to αVβ3.

**Figure 3:**
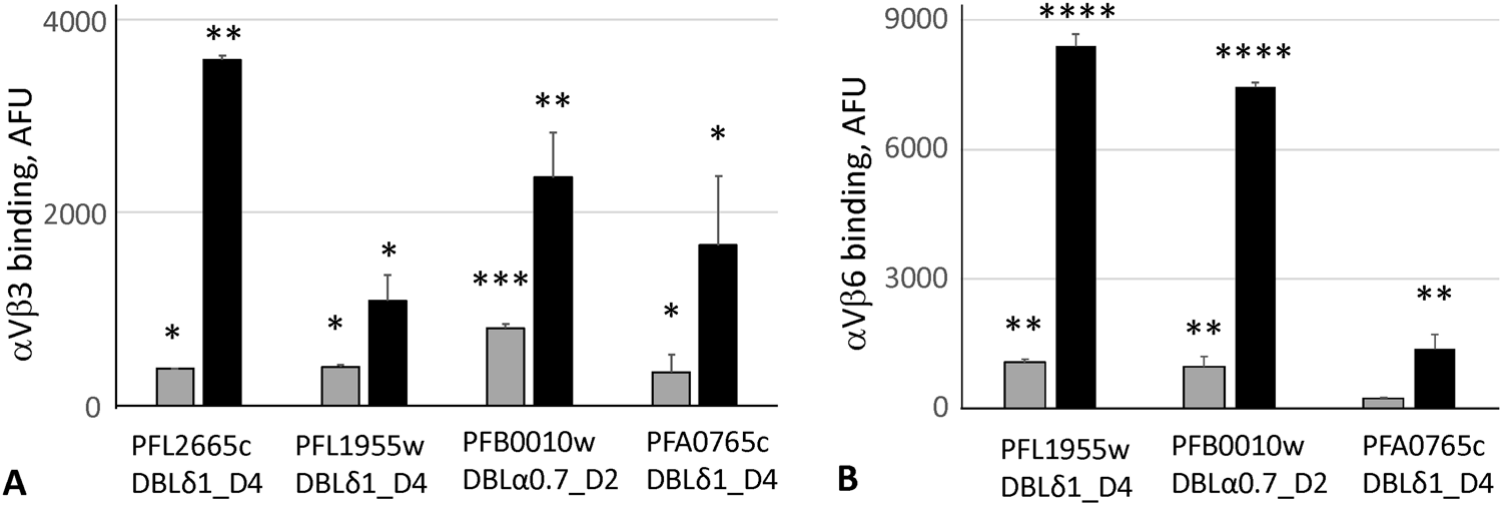
Binding of bead-immobilized single DBL domains containing RGD motif to αVβ3 (**A**) and αVβ6 (**B**) integrins at two concentrations. Gray bars, 1 μg/ml; black bars, 5 μg/ml. AFU, Arbitrary fluorescence units. Control value for HisAdEx construct was subtracted from binding of domain constructs. Bars represent means of duplicate measurements. Error bars indicate Standard Deviations (SD). Differences in binding between each domain and control construct for each concentration were calculated by one-way ANOVA using Holm-Sidak’s multiple comparison tests. *p < 0.05, **p < 0.01, ***p < 0.001, ****p < 0.0001. These experiments were repeated at least twice with similar qualitative results.

These data clearly indicate that αVβ3 and αVβ6 integrins and the DBLδ_D4 domain from PFL2665c may organize receptor-ligand pairs, through which IE can potentially interact with endothelial and immune cells expressing this receptor. Interestingly, this DBL domain belongs to the delta class of domains^18^. Based on disproportionate distribution of RGD sequence in DBL domains, the DBLα0 class of domains has been predicted to likely interact with RGD-binding integrins. However, we did not find any binders among three DBLα0 class domains, which we tested as DBL-CIDR tandems (Fig. 1). Although all DBLα0-CIDR tandems used in our work were able to bind CD36 through CIDRs (Supplementary Fig. 2), false negatives due to potential misfolding cannot be excluded. It is also possible that presence of the CIDR domain in tandem may affect binding of integrin by DBL domain in some of the tandem constructs. Therefore, we tested three additional well-expressed in our hands single DBL domains containing an RGD motif, including two gamma-class domains (PFA0765c DBLδ1_D4 and PFL1955w DBLδ1_D4) and one alpha-class domain, PFB0010w DBLα0.7_D2, which was the part of the inactive tandem tested (Fig. 1). The results, shown in Fig. 3, demonstrate that all these domains bind both αVβ3 and αVβ6 integrins. These data experimentally confirm that DBLα0 domains can bind RGD-binding integrins and expand the number of DBLδ1 domains, in addition to PFL2665c DBLδ1_D4, that can bind these integrins. Because PFB0010w DBLa0.7_D2 binds integrins while DBLα0.7_D2-CIDRα2.2_D3 does not, there may be an effect of the neighboring CIDR domain on integrin binding, which may have some biological sense, or might be simply an artefact of the recombinant constructs. Definitely, these *in vitro* data should be further confirmed using live IE in future work. In this work, the experimental identification (using both bead-bound domains and live IE) of the first, to the best of our knowledge, αVβ3 and αVβ6 integrin-binding PfEMP1 domain (DBLδ1_D4) may further contribute to better understanding of malaria-host interactions.

As integrins are important players in the physiology of endothelial and immune cells, it is likely that specific cellular functions might be affected or disturbed by their interactions with IE. Interestingly, though irrelevant to PfEMP1 proteins, non-RGD-binding integrin αDβ2 may mediate experimental malaria-associated acute respiratory distress syndrome in mice^32^, and αV-containing integrins may strongly bind to a *P*. *falciparum* sporozoite surface proteins TRAP^33^. Our identification of an IE ligand for the integrin receptors further expands our knowledge about malaria interactions with an important class of host receptors, integrins, and allows studying of these effects at the molecular level. Future experiments with host vascular and immune cells expressing these integrins may reveal further physiologically relevant details of PfEMP1::integrin binding process.

In our example, PFL2665c molecules can bind two different receptors (CD36 and integrin) on the surface of the same cell, as both these receptors are normally expressed in endothelial cells and cells of the immune system, including monocyte/macrophages. PFL2665c resembles, in this sense, Group A PfEMP1 proteins that have binding ability to two different receptors, for example ICAM-1 and EPCR by PF11_0521, through two neighboring domains, CIDRα1.4_D3 and DBLβ3_D4^14^. Moreover, binding of IE to CD36 recruits αVβ1 integrin in microvascular endothelial cells to increase strength of binding^34^. If similar effects also occur with other integrins, for example αVβ3 and αVβ6, this may potentially contribute to the level of virulence (positively or negatively) of some CD36-binding parasite lines with double binding specificity.

Our work also assigns a functional role for DBL domains from the C-terminal half of the most numerous 4-domain class of PfEMP1 proteins. It is logical to suggest that other domains of the C-terminal half of PfEMP1 protein may have similar function interacting with other, not yet identified receptors.

In conclusion, we have identified a novel αVβ3 and αVβ6 integrin::DBLδ1_D4_PFL2665c_ PfEMP1 domain pairs that may contribute to IE cytoadhesion to endothelial and immune cells and affect the physiological state of these cells, which, in turn, may play a role in malaria pathology.

## Methods

### Parasite cultures

Selection of the NF54 line E9 by limiting dilution, which expresses the single *var* gene, PFL2665, has been described earlier^23^. The aliquots of this line (expanded in 10–15 growth cycles and frozen) were thawed and grown in human O+ erythrocytes at 2% hematocrit in complete RPMI 1640 medium supplemented with 10% human serum and 40 μg/mL gentamicin sulfate for about 2–5 growth cycles (as described in^31^) before binding experiments. Parasite lines CS2, NF54 and 3G8 (kindly provided by Dr J. Smith) were cultured similarly. Cultures were maintained at 37 °C in a gas mixture of 5% CO2, 5% O2 and 90% N2.

### Binding PfEMP1 domains to αVβ3 and αVβ6 integrins

The following domains were cloned into pHisAdEx vector, expressed, and purified/immobilized on Bio-Plex beads using methods described earlier^35^: DBLα1.2_D2-CIDRα1.1_D3_**PFD0020c**_, DBLα0.7_D2-CIDRα2.2_D3_**PFB0010w**_, DBLα0.19_D2-CIDRα2.3_D3_**PFL2665c**_, DBLδ1_D4-CIDRβ1_D5_**PFL2665c**_, DBLα0.16_D2-CIDRα3.4_D3_**PFL1955w**_, DBLβ3_D3_**PF11_0521**_, DBLα0.9_D2-CIDRα2.2_D3_**PFL0005w**_, DBLβ3_D3_**PF13_0003**_, DBLα2_D2-CIDRα1.6_D3_**PF08_0140**_, CIDRα3.1_D3_**PF08_0106**_, DBLζ5_D6_**PF08_0141**_, DBLα0.17_D2-CIDRα3.1_D3_**PF07_0049**_, DBLα0.24_D2-CIDRα3.4_D3_**PFD1015c**_, DBLα0.20_D2_**MAL7P1.56**_, DBLβ5_D4_**PFL0020w**_, DBLα0.7_D2_**PFB0010w**_, DBLδ1_D4_**PFL1955w**_, and DBLδ1_D4_**PFA0765c**_ (domain classification according to^18^). PCR primers for each construct and protein domain boundaries are shown in Supplementary Fig. 7. All constructs were verified by sequencing. Each construct is fused with Green Fluorescence Protein at the C-terminus and the green fluorescence indicates expression of the full-length protein. Integrin receptors αVβ3 (R&D Systems, Cat #3050-AV) and αVβ6 (R&D Systems, Cat #3817-AV) were biotinylated and their binding to bead-bound PfEMP1 domains (1000 to 2000 beads in the reaction) was measured in duplicates on BioPlex 200 machine (BioRad) as described before^23^ using concentrations of the biotinylated receptor indicated in the text and figure legends. As the negative control in these experiments, beads with immobilized HisAdEx construct^8,21^, which contains all the same parts as recombinant domain constructs but short irrelevant 37-amino acid long peptide instead of PfEMP1 domain, were used. Experiments on receptor binding to bead-immobilized domains were repeated at least 3 times with qualitatively similar results. Results of representative experiments are shown.

### Determination of αVβ3 or αVβ6 integrin:: DBLδ1_D4_PFL2665c_ interaction avidity constants

Measurements and calculations were performed as described in our earlier publication^22^ for ICAM1::PF11_0521 DBLβ3_D4 interactions with the following modifications. We used 1xTris-buffered saline (TBS) supplemented with 1 mM CaCl_2_ and 1 mM MgCl_2_ for all incubations and streptavidin-PE as the detection molecules. About 1000 to 2000 beads with immobilized PFL2665c DBLδ1_D4 domain were incubated with 3 concentrations (3, 6, and 12 µg/ml) of biotinylated integrin at 0, 10, 30 min (αVβ3), and at 0, 5, 15 min (αVβ6) times in two independent experiments for each receptor. Each experiment was performed in duplicates for αVβ3 and in duplicate (exp. 1) and triplicate (exp. 2) for αVβ6. Binding was measured using BioPlex 200 machine (BioRad), corrected for the control construct HisAdEx^8,21^, initial velocities calculated, and plotted using Lineweaver-Burk plots to determine equilibrium dissociation constant K_D_. Molecular weight (MW) of soluble recombinant αVβ3 and αVβ6 was 191.3 kDa and 189.1 kDa, respectively, according to manufacturer information for molar concentration calculations. Linear regression lines, standard error of means (SEM) error bars, and p-values for each regression were calculated using GraphPad Prizm software.

### Infected erythrocyte adhesion and adhesion-inhibition assays

Binding of IE lines to various receptors (CSA from Sigma cat # C9819, and ICAM1, CD36, αVβ6, and αVβ3 – all from R&D Systems, cat ## 720-IC, 1955-CD, 3817-AV, 3050-AV, respectively) or control (BSA) immobilized on Petri dishes were performed using previously published methods and media supplemented with 1 mM CaCl_2_ and 1 mM MgCl_2_^23,36^. Before the adhesion assays, IE in trophozoite stage were enriched by magnetic LD columns (Milteni Biotec, cat#130-042-901) as described by manufacturer. Percentage of mature trophozoites in the elution varied from 22 to 95% and was adjusted using uninfected erythrocytes to the levels specified in the appropriate Figure legends. Cyclic peptide antagonist cyloRGDFV (Sigma Aldrich, #SCP0111) was used to inhibit binding of IE to αVβ3 at concentrations specified in Figure legends. Enriched trophozoites were incubated with inhibitor peptide for 30 min at 37 °C, placed over the immobilized receptor, and incubated for 30 min at 37 °C. Non-adherent cells were washed by 1xTBS/1 mM CaCl_2_/1 mM MgCl_2_. Adherent cells were counted using microscopy in 20–40 randomly selected microscope fields. Each test was performed in duplicate spots of receptor. Binding was presented as averaged number of bound IE per filed and Standard Error of Mean (SEM) re-calculated for binding per mm^2^. Each binding experiment was repeated at least 2 times. Results of typical experiment in each group are presented.

Anti-CD36 monoclonal antibody FA6-152 (Abcam, ab17044) at 5 μg/ml was used to inhibit E9 binding to CD36 immobilized on Petri dish at 10 μg/ml using approach described above.

### Ethics statement

All methods were carried out in accordance with relevant guidelines and regulations. Ethics approval was obtained from the Florida Atlantic University Institutional Review Board committee for using human erythrocytes for culturing malaria parasites. Blood was purchased from Valley Biomedical.

## Electronic supplementary material

Supplementary Information

## Data Availability

All data generated or analyzed during this study are included in this published article (and its Supplementary Information files).
