# Supplementary material for: Plasmodium falciparum infected erythrocytes can bind to host receptors integrins αVβ3 and αVβ6 through DBLδ1_D4 domain of PFL2665c PfEMP1 protein
Source: Sci Rep. 2018 Dec 14;8:17871. doi: 10.1038/s41598-018-36071-2 (PMC6294747; doi:10.1038/s41598-018-36071-2)
Supplement: Supplementary file 1 — Supplementary Information [file 41598_2018_36071_MOESM1_ESM.pdf]

Supplementary Information

Plasmodium falciparum infected erythrocytes can bind to host receptors integrins αVβ3 and αVβ6 through DBLδ1_D4 domain of PFL2665c PfEMP1 protein

Olga Chesnokov[1, #], Jordan Merritt[1, #], Sergey O. Tcherniuk[1], Neta Milman[2], Andrew V. Oleinikov[1,*]

Supplementary Figure 1. Location of RGD motifs in predicted secondary structure of PFL2665c DBL2 domain aligned with PfEBA175 EF1 DBL and EF2 DBL secondary structures for comparison.

```
DBL2       ---------gddlkqacstkygpkaptswkcvpsgvstatsgeggdaksrerreagvpta    51
EF1        --grqt-ssnnevlsnc---rekrkgmkwdckk----------------------------    27
EF2        kekrehidlddfskfgcdknsvdtntkvweckk----------------------------    33
                    .:     *           *.*
                           1             2

                                 HELIX 1
DBL2       tssgnttgggkdgatggsicvpprrrrlyvggltkwakkytgntgesksqegvlqtkavv    111
EF1        ----------knd-rsnyvcipdrriqlcivnlaiiktyt--------------------    56
EF2        ----------pyklstkdvcvpprrqelclgnidriydkn--------------------    63
                             :*:* **  .*  :  .:
                              3        4

DBL2       dgkanaegggqkgargpnggtegansgkgaqqqqeqqqeqqqeqqqqqqqqqqqphstd    171
EF1        ------------------------------------------------------------    56
EF2        ------------------------------------------------------------    63


                             HELIX 2
DBL2       ssssppssnprdvdlrnafvesaaietfflwdrykkekekekedieknqqdtvaytssve    231
EF1        -----------ketmkdhfieaskkesqlllkkndnk-----------------------    82
EF2        -----------llmikehilaiaiyesrilkrkyknk-----------------------    89
                         ::: ::  :  *: :*  : .::

                            HELIX 3                        HELIX 4
DBL2       kdpqeelqrgdipdgflrqmfytlgdyrdilysgdkengnky-mlvddikdisdkiksil    290
EF1        -----------ynskfcndlknsfldyghlamgndmdfggystkaenkiqevfkgahge-    130
EF2        -----------ddkevckiinktfadirdiiggtdywndlsnrklvgkintnsnyvh---    135
                        . . . :  :: *  .:  . *   .       ..*:   .
                           5

                  HELIX 5
DBL2       nsdvgqkttakqwwddngqhiwngmiyaltyntdtpsgdkptqidevraqlwdekekkpk    350
EF1        isehkiknfrkkwwnefreklweamlsehknninn-------------------------    165
EF2        rnkqndklfrdewwkvikkdvwnviswvfkdktv--------------------------    169
            ..   *   .:**.   :.:*: :     . :

                                                                HELIX 6
DBL2       ktdndhdytyenvelkedddqsgaktpsassgsndpinnpqlsdfveiptyfrwlhewgs    410
EF1        --------------------------------------ck--nipqeelqitqwikewhg    185
EF2        --------------------------------------ckeddien-ipqffrwfsewgd    190
                                                          . :*: ** .
                                                 6

             HELIX 6 (cont)                                 HELIX 7
DBL2       dfcgtrkrmlgkikhecrgd---kvcsgygencddqlkdnpsifpslncpscgtpcryykkwi 470
EF1        efllerdnraklpkskcknnalyeac----------------------ekecidpcmkyrdwi 226
EF2        dycqdktkmietlkveckekp----c---------------------eddnckrkcnsykewi 228
           ::   : .      * :*: .     *                     .*   *   *:.**
             7            8        9                        10  11

             HELIX 7 (cont)                   HELIX 8
DBL2       ntkkteydkqksayeqqqgkcekenngaegndhdkkfctri-qncneakdflktlgpcrt    529
EF1        irskfewhtlskeyetqkvpke----------naenylikisenkndakvs-lllnncda    275
EF2        skkkeeynkqakqyqeyqkgnnykmysefksikpevylkkysekcsnlnfedefkeelhs    288
             .* *:..  . *:  :   :          : : :  :   : .: :          :
                                                      12

        HELIX 9
DBL2       ndesg-----   534
EF1        eyskycd-ck   284
EF2        dyknkctmcp   298
           : ..
               13 14
```

red – alpha HELIX

blue – extended strand

green highlight – conserved Cys

Pink highlight – RGD motif

DBL2 PFL2665 secondary structure was predicted at https://npsa-prabi.ibcp.fr/cgi-bin/secpred_consensus.pl according to Combet et al., TIBS 2000, V. 25, No 3 [291]:147-150

PfEBA175 EF1 DBL and EF2 DBL secondary structure, HELIX and Cys numbering indicated according to Hodder et al., JBC 2012, V. 287, 32922-32939.

Second RGD is located in a small loop arranged by 2 surrounding cysteine residues (Cys 8 and 9), involved in disulfide boding with other Cys residues according to the model shown in Singh, S. K., et al. Nature 2006, 439(7077): 741-744.

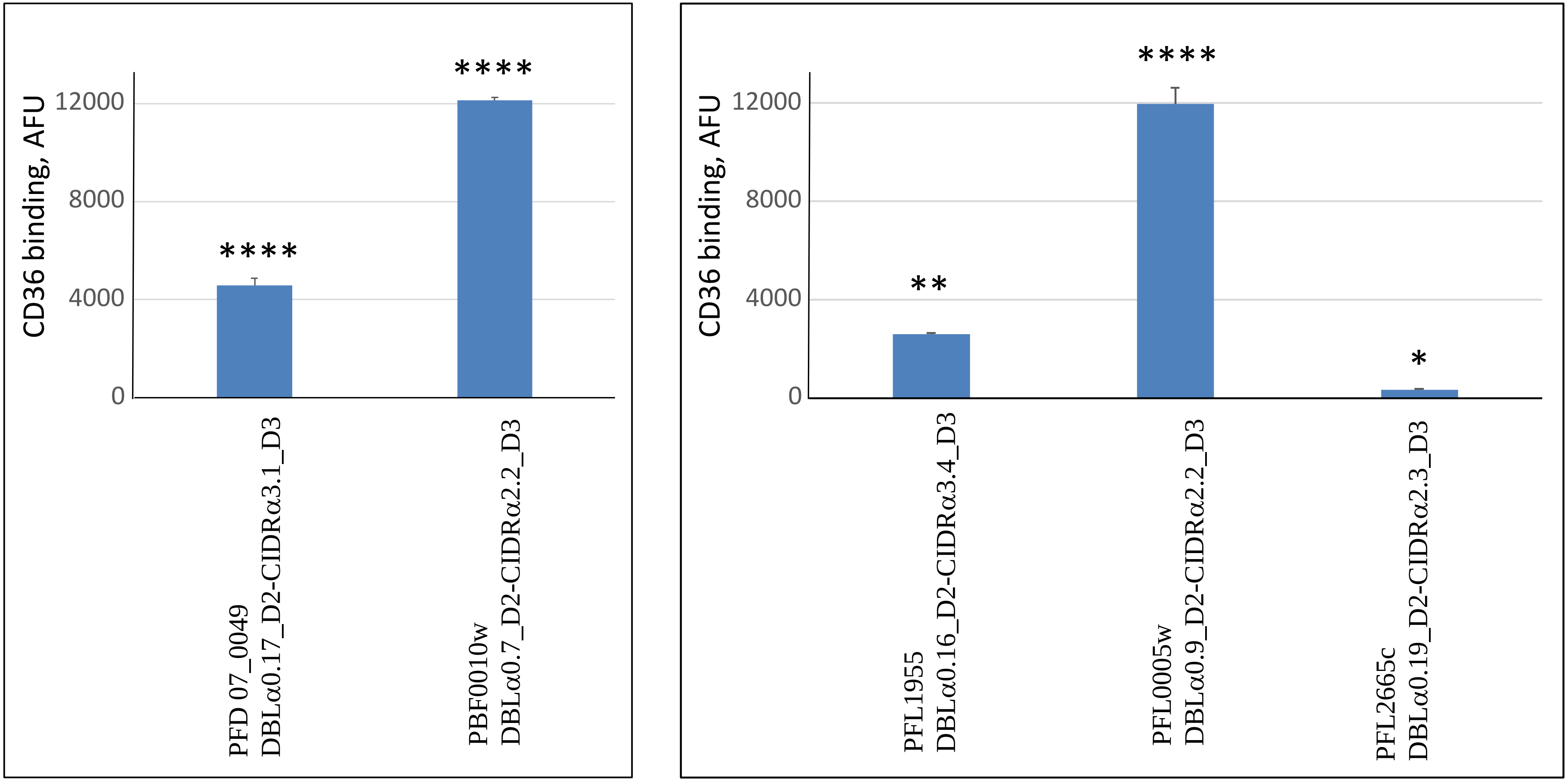

Supplementary Figure 2. CD36 binding to tandem domains. BioPlex beads with immobilized tandem domains and control construct HisAdEx were incubated with 5 μg/ml soluble CD36 for 1h at room temperature. AFU, Arbitrary fluorescence units. Control construct value was subtracted from binding of each domain construct. Bars represent means of duplicate measurements. Error bars indicate Standard Deviations (SD). Differences in binding between each domain and control construct for each concentration were calculated by one-way ANOVA using Holm-Sidak's multiple comparisons tests. *$p<0.05$, **$p<0.01$, ****$p<0.0001$. Measurements performed in the same experiment presented in a single box. These experiments were repeated twice with similar qualitative results.

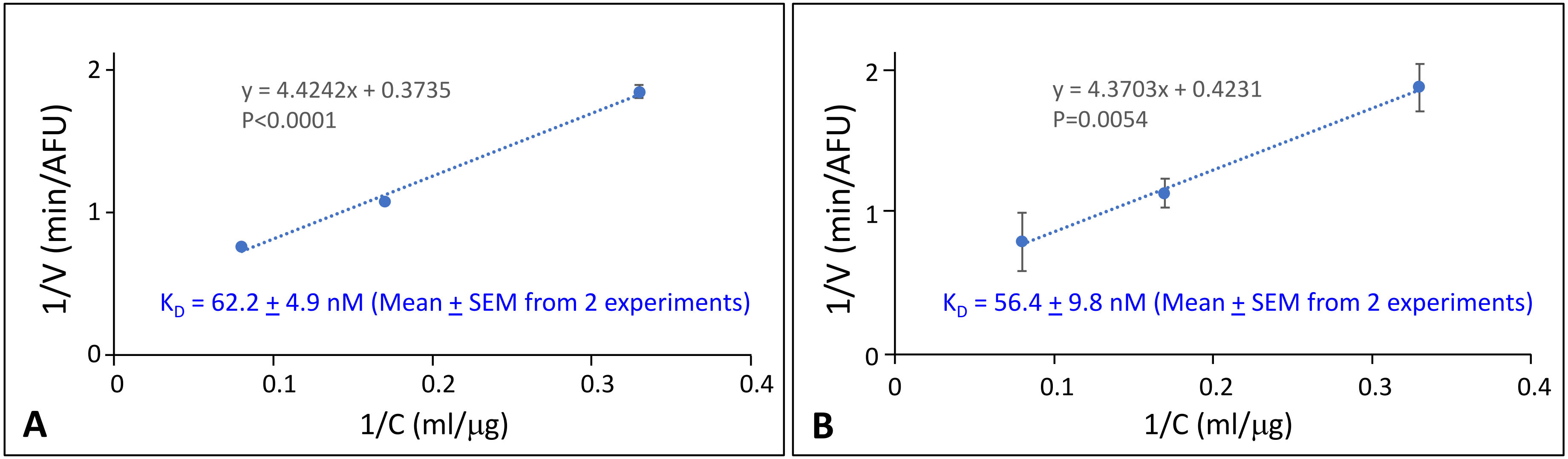

Supplementary Figure 3. Determination of equilibrium dissociation constant KD for integrins αVβ3 (A) and αVβ6 (B) binding to PFL2665 DBLδ1_D4 domain. Kinetics of binding were measured for each receptor in two independent experiments. Error bars are standard errors of mean from two independent experiments. Lines and formulas show linear regressions and p-values of each regression calculation. V, initial velocity of binding; C, concentration of integrin; min, minutes; AFU, arbitrary fluorescence units.

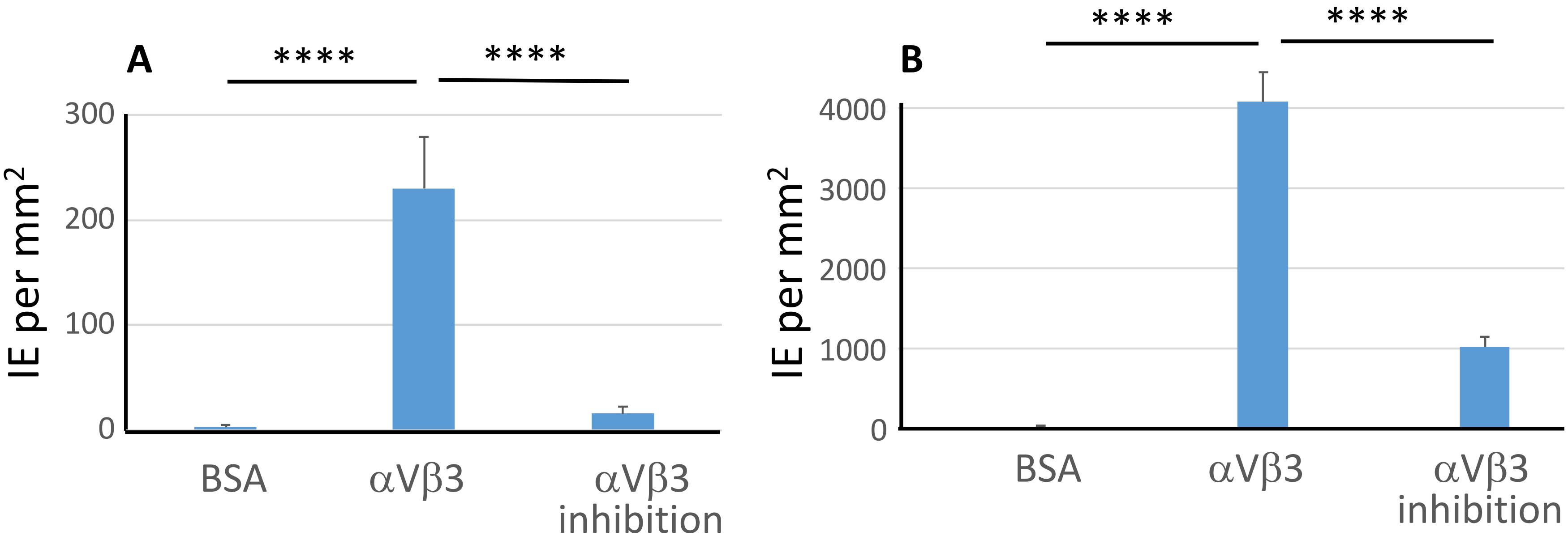

Supplementary Figure 4. Specific binding of E9 line to surface-immobilized αVβ3 integrin. Binding of E9 line (0.5% hematocrit and 2% parasitemia (**A**) or 35% parasitemia (**B**)) to surface-immobilized αVβ3 integrin (10 μg/ml) was inhibited by 10 μg/ml (**A**) or 25 μg/ml (**B**) of soluble αVβ3 pre-incubated with the parasite line for 30 min at 37^0C before incubation with the immobilized integrin. Binding was measured by counting attached infected erythrocytes in 32 - 40 microscope fields. Bars indicate Means and Error bars indicate Standard Error of Mean (SEM). P values obtained by Holm-Sidak's multiple comparisons test (ANOVA). ****$P<0.0001$. Similar qualitative results obtained at wide range of parasitemia.

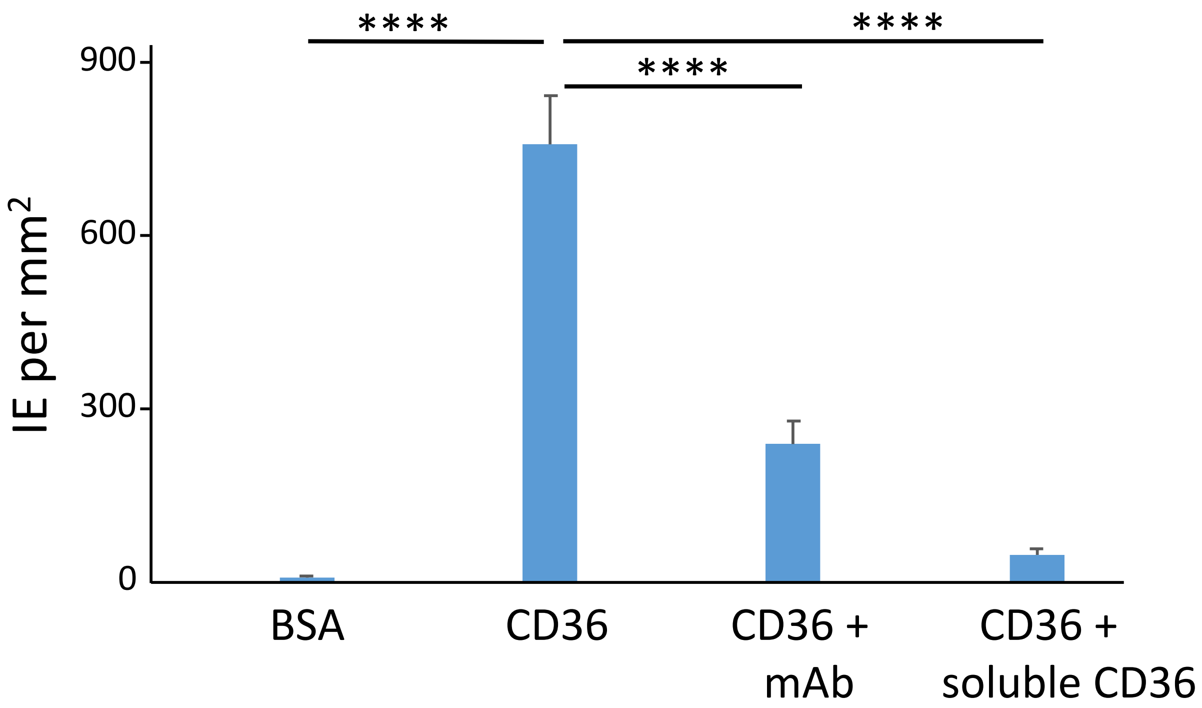

Supplementary Figure 5. Specific binding of E9 line to surface-immobilized CD36. Binding of E9 line (0.5% hematocrit and 35% parasitemia) to surface-immobilized CD36 (10 μg/ml) was inhibited by 5 μg/ml of anti-CD36 mAb FA6-152 or by 25 μg/ml of soluble CD36. mAb was pre-incubated with the receptor and soluble CD36 was pre-incubated with the parasite line, respectively, for 30 min at 37^{0}C before incubation of E9 with the immobilized CD36. Binding was measured by counting attached infected erythrocytes in 18 - 30 microscope fields. Bars indicate Means and Error bars indicate Standard Error of Mean (SEM). P values obtained by Holm-Sidak's multiple comparisons test (ANOVA). ****$p<0.0001$. Experiment was repeated 2 times with qualitatively similar results.

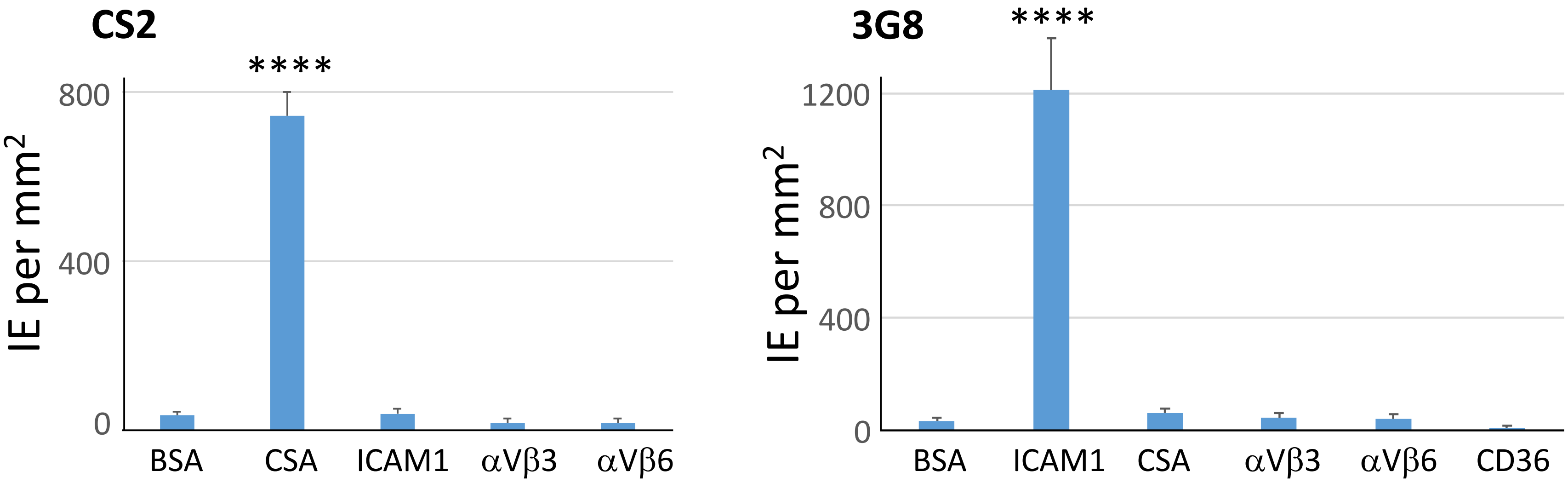

Supplementary Figure 6. Parasite lines CS2 and 3G8 that bind to CSA and ICAM1 receptors, respectively, do not bind to integrins αVβ3 and αVβ6. Binding was performed at 0.5% hematocrit and 4% parasitemia (**CS2**) or 3% parasitemia (**3G8**) to surface-immobilized BSA (3%), CSA (100 μg/ml), ICAM1 (10 μg/ml), αVβ3 (10 μg/ml), αVβ6 (10 μg/ml), and CD36 (10 μg/ml). Binding was measured by counting attached infected erythrocytes in 15 - 18 microscope fields. Bars indicate Means and Error bars indicate Standard Error of Mean (SEM). P values obtained by Holm-Sidak's multiple comparisons test (ANOVA). ****$P<0.0001$. Experiment was repeated 2 times for CS2 and 3 times for 3G8 with qualitatively similar results.

Supplementary Figure 7. PfEMP1 constructs cloned into pHisAdEx vector (with two indicated exceptions cloned into pAdEx) for expression and functional testing. Pairs of primers with their names used to amplify the PfEMP1 domains inserted in the vector are shown below. Restriction site used for cloning is underlined.

PFD0020c DBLα1.2_D2-CIDRα1.1_D3 (AA 2-745)

F-**PFD0020c**-DBL1-CIDR-Bam
CccGG ATCCagGGGACAGGTTCATCAACTCCTTCGG

R-**PFD0020c**-DBL1-CIDR-BspE
CccTCC GGAGCCACCATTATTGTTTGTTCCACA

PF13_0003 DBLβ3_D3 (AA 744-1204)

F-Bam-**PF13_0003**-DBL2
CccGGATCCagCCACGCGCCCATAATCCGTGTG

R-Eco-**PF13_0003**-DBL2
CccGAATTCcTTTCCGTACCTTCGTCTTCTGTC

PF11_0521 DBLβ3_D3 (AA 728-1249)

F-Bam-**PF11_0521**-DBL2
CccGGATCCagAACCCGTGTGCTAAACCTCATGG

R-Eco-PF11_0521-DBL2
CccGAATTCcCGGATTTGGAGTAGAAGGAGG

PF08_0141 DBLζ5_D6 (AA 1700-2077)

F-Bam-**PF08_0141**-DBL4
CccGGATCCagCCTTCTATTGAAGAATGTGATATCAA

R-Eco-**PF08_0141**-DBL4
CccGAATTCcATCCAATGGATCAAAATCTTTCGCAC

PFB0010w DBLα0.7_D2-CIDRα2.2_D3 (AA 2-837)

F-Bam-**PFB0010w**-DBL1
CccGGATCCagGCGACTGGTAGTGGGGGCG

R-Xba-**PFB0010w**-CIDR1
CccTCTAGACGTGTCCGCGAGTGCGGTTTTC

PFL2665c DBLα0.19_D2-CIDRα2.3_D3 (AA 2-858)

F-Bam-**PFL2665c**-DBL1
CccGGATCCag GGGGGAAGTAATGGCGGTGG

R-Eco-**PFL2665c**-CIDR1
CccGAATTCcTACATCGTCTGTTGGTACCGGTTC

PFL2665c DBLδ1_D4-CIDRβ1_D5 (AA 870-1766)

F-Bam-**PFL2665c**-DBL2
CccGGATCCagGGTGACGATCTCAAACAAGCCTG

R-Eco-**PFL2665c**-CIDR2
CccGAATTCcACCAGCTGGAGCAGGTTCGG

PFL1955w **DBLα0.16_D2**-CIDRα3.4_D3 (AA 2-899) cloned in pAdEx vector

F-Bam-**PFL1955w**-DBL1
CccGGATCCagGCGCCATCCACTACATACAGTAG

R-Eco-**PFL1955w**-CIDR1
CccGAATTCcTGCGACTTCTTTAAATTGTTCGGGG

PFL0005w DBLα0.9_D2-CIDRα2.2_D3 (AA 2-846)

F-Bam-**PFL0005w**-DBL1
CccGGATCCagGGGCCCCAGCCGGCTGTG

R-Eco-PFL0005w-CIDR1
CccGAATTCcTGCATCTTCAAAATTTTTGGTGCTC

PF08_0140 DBLα2_D2-CIDRα1.6_D3 (AA 2-743) cloned in pAdEx vector

F-**PF08_0140**-DBL1-CIDR-Bam
CccGGATCCagGTTCCCCCTGTCCGTTCCCCTCG

R-**PF08_0140**-CIDR-Eco
CccGAATTCcACCATTATTGTTTTTTCCACAGGGG

PFL0020w DBLβ5_D4 (AA 750-1242)

F-Bam-**PFL0020w**-DBL2
CccGGATCCagAATCCGTGTAGCGCCCAACCTGG

R-Eco-**PFL0020w**-DBL2
CccGAATTCcCGCATCTTTTTTTTCTTCTTCCTTC

PF08_0106 CIDRα3.1_D3 (AA 406-893)

F-Afl-**PF08_0106**-CIDR1
CccCTTAAGAAGGTAGCATCAGGTAGTAGTAGG

R-Eco-**PF08_0106**-CIDR1
CccGAATTCcGTCGTCTTTAAATAGTTTGTCCACTATGTC

PF07_0049 DBLα0.17_D2-CIDRα3.1_D3 (AA 2-920)

F-Bam-**PF07_0049**-DBL1
CccGGATCCagGGGCAAAAAGTGTACGAAGAAATAGTGGG

R-Eco-**PF07_0049**-CIDR1
CccGAATTCcTTCTTCCTTTTGGTCGACACGTGGTG

PFD1015c DBLα0.24_D2-CIDRα3.4_D3 (AA 2-813)

F-Bam-**PFD1015c**-DBL1
CccGGATCCagGCGCCGCAAAGTAGTGGGGGTAGTG

R-Eco-**PFD1015c**-CIDR1
CccGAATTCcGTCGTCGCCACTACCAGTGGAGG

MAL7P1.56 DBLα0.20_D2 (AA 2-416)

F-Bam-**MAL7P1.56**-DBL1
CccGGATCCagGCGCGAGATCCTCGTGGTGGGGG

R-Eco-**MAL7P1.56**-DBL1
CccGAATTCcTGGTGCTCCCTCTGTATATATTTTTATTTC

PFB0010w DBLα0.7_D2 (AA 2-436)

F-Bam-**PFB0010w**-DBL1
CccGGATCCagGCGACTGGTAGTGGGGGCG

R-Xba-**PFB0010w**-DBL1
CccTCTAGAGCCTTCATTTCGTAGTTCTTC

PFL1955w DBLδ1_D4 (AA 897-1353)

F-Bam-**PFL1955w**-DBL2
CccGGATCCagGAAGTCGCATGTAAACAAAAATATG

R-Eco-**PFL1955w**-DBL2
CccGAATTCcATATATTTTTTCTTGTTTTTCATATTCCGC

PFA0765c DBLδ1_D4 (AA 903-1343)

F-Bam-**PFA0765c**-DBL2
CccGGATCCagACCGACCAGACTAATCTCACAAAGG

R-Eco-**PFA0765c**-DBL2
CccGAATTCcAGTTTTACATGGTCTTAACGTTTGTAAG
